# Effect of patient‐centered self‐management intervention on glycemic control, self‐efficacy, and self‐care behaviors in South Asian adults with type 2 diabetes mellitus: A multicenter randomized controlled trial

**DOI:** 10.1111/1753-0407.13611

**Published:** 2024-09-12

**Authors:** Kainat Asmat, Erika Sivarajan Froelicher, Khairunnisa Aziz Dhamani, Raisa Gul, Nazeer Khan

**Affiliations:** ^1^ Faculty of Nursing and Midwifery Shifa Tameer‐e‐Millat University Islamabad Pakistan; ^2^ Department of Physiological Nursing, School of Nursing University of California San Francisco California USA; ^3^ Department of Epidemiology & Biostatistics, School of Medicine University of California San Francisco California USA; ^4^ The Office of Research, Innovation and Commercialization (ORIC) Shifa Tameer‐e‐Millat University Islamabad Pakistan

**Keywords:** behavioral training, counseling, glycemic control, patient‐centered care, randomized controlled trial, self‐management, type 2 diabetes mellitus

## Abstract

**Background:**

This study aimed to test the efficacy of patient‐centered self‐management intervention (PACE‐SMI) to improve HbA1c, self‐efficacy, and self‐care behaviors in adults with type 2 diabetes mellitus (T2DM).

**Methods:**

In this multicenter, parallel two‐arm randomized controlled trial, 612 adults with T2DM and HbA1c ≥ 7% were enrolled and assigned to the control group (*n* = 310) and the intervention group (*n* = 302) using stratified permuted block randomization. The control group received usual care, whereas the intervention group received usual care plus nurse‐led, theory‐driven, culturally tailored PACE‐SMI, comprising eight weekly sessions of individualized education, counseling, behavioral training, and home visit. Outcomes were assessed at baseline, postintervention, and 3 months follow‐up.

**Results:**

Data at 3 months were provided by 583 participants (control: *n* = 295, intervention: *n* = 288). Per‐protocol analysis showed that the intervention group had a lower mean HbA1c (8.49% [standard deviation (SD), 1.58]) than the control group (8.74% [SD, 1.62]), with small yet statistically significant mean difference of 0.25% (95% confidence interval [CI], −0.01 to 0.51; Cohen's *d* = 0.16; *p* = 0.03). Self‐efficacy and self‐care behaviors significantly improved in the intervention group (116.89 [SD, 25.50] and 70.01 [SD, 17.97]) compared to the control group (75.43 [SD, 18.99] and 51.54 [SD, 12.04]), with mean differences of 41.48 (95% CI, 37.83–45.13; Cohen's *d* = 1.84; *p* < 0.0001) and 18.56 (95% CI, 16.08–21.04; Cohen's *d* = 1.22; *p* < 0.0001), respectively. Linear regression analysis indicated the effect of PACE‐SMI on HbA1c was significantly mediated by improvements in self‐efficacy and self‐care behaviors (*R* ^2^ = 0.232, *p* < 0.001).

**Conclusion:**

PACE‐SMI led to modest but significant improvement in HbA1c and substantial enhancements in self‐efficacy and self‐care behaviors in adults with T2DM.

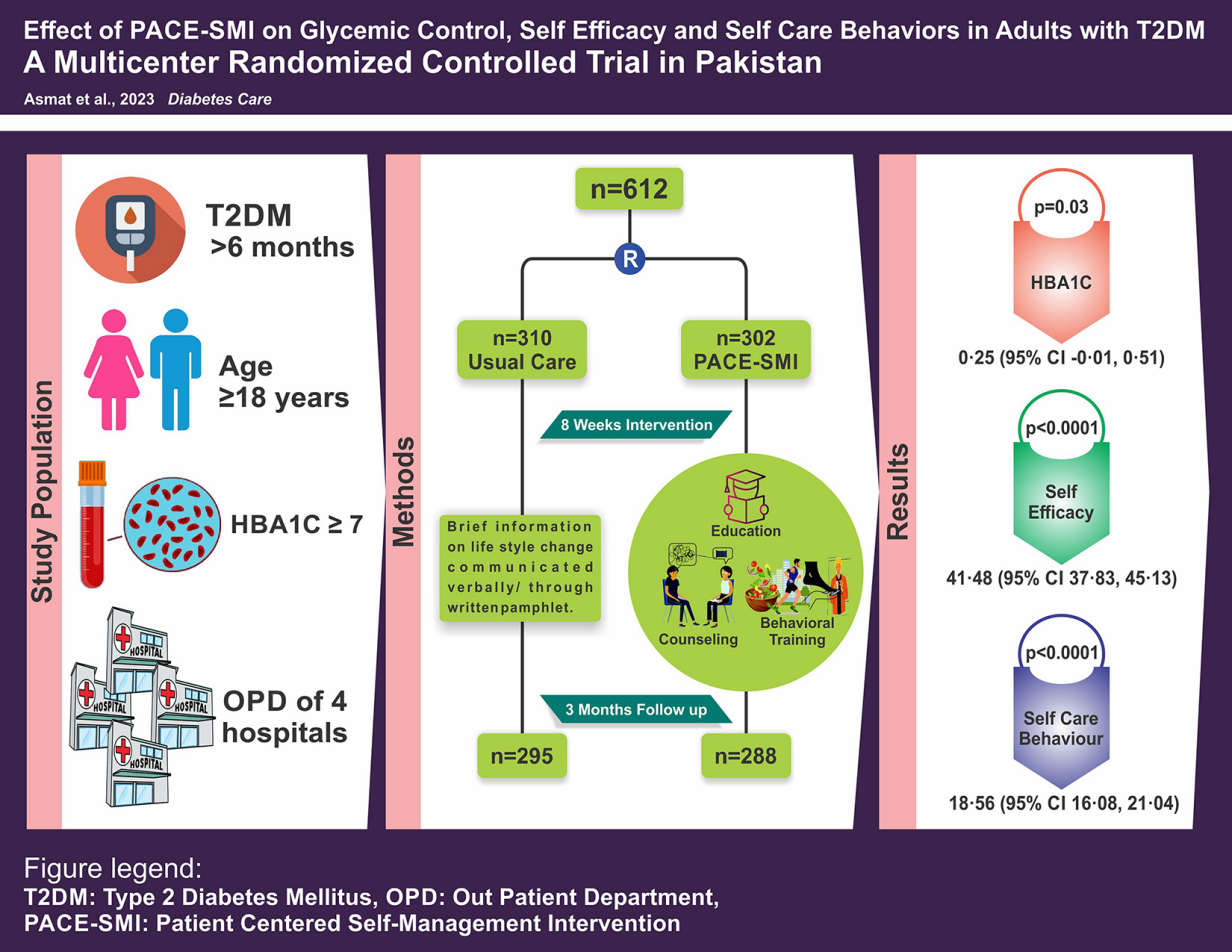

## Graphical abstract

Highlights

- T2DM dominates worldwide, especially in the low middle income countries (LMICs) of South Asia.
- Despite acknowledging self‐management as the corner stone in T2DM, LMICs face an implementation gap due to lacking policies and the fragmented care systems, neglecting crucial personal, behavioral, and social factors.
- In this first large‐scale multicenter randomized controlled trial conducted in Pakistan, a LMIC, the nurse‐led PACE‐SMI demonstrated significant improvement in HbA1c, self‐efficacy, and self‐care behaviors in adults with T2DM at 3 months.
- Considering the substantial health and economic impact of T2DM in LMICs, evidence presented in this study holds implications for the healthcare policies, advocating for integration of PACE‐SMI into standard practice.

## 1 INTRODUCTION

Type 2 diabetes mellitus (T2DM) accounts for over 90% of DM cases worldwide, with a majority of the affected population living in the low middle income countries (LMIC) of South Asia.^1^ Pakistan, a LMIC with an estimated 233 million population, ranks third in the world with the highest number of adults having DM (mostly T2DM).^2^ Currently, 33 million adults in Pakistan have T2DM, while an additional 11 million have impaired glucose tolerance and are at risk of developing T2DM.^3^ Correspondingly, over a quarter (26.9%) adults with T2DM are undiagnosed and are at risk for serious complications, that is, kidney failure, heart attack, stroke, blindness, and lower limb amputation.^4^ The rising prevalence of T2DM and associated complications have led to a reduction in functional capacity of the individuals, resulting in impaired quality of life, and elevated healthcare costs with significant implications for the country's health care system and the economy.^5^, ^6^ Given the substantial health and economic impact of T2DM in LMICs such as Pakistan, there is an urgent need for effective interventions to halt the disease progression and lower the risk of associated complications. The rising burden of T2DM in Pakistan is driven by aging population, increasing urbanization with sedentary lifestyle, unhealthy dietary habits, and obesity.^7^ The evidence suggests that the key to slow down T2DM progression and reduce the risk of serious complications is lifestyle modification, also known as self‐management.^8^, ^9^, ^10^ Literature from the developed countries reveals that self‐management education and support in behavioral modification empowers individuals with T2DM to effectively manage their condition, leading to better health outcomes and reduced healthcare costs.^11^, ^12^, ^13^, ^14^ However, integration of such care components in the healthcare system of a LMIC such as Pakistan, still remains a challenge. A few previous studies have been conducted in relation to self‐management education, but none focused on counseling and behavioral skills training.^15^, ^16^, ^17^ Although education is undoubtedly important, it alone is insufficient to address complex condition like T2DM, influenced significantly by personal, behavioral, and social factors. Therefore, a patient‐centered behavioral or social intervention may have a lasting impact on effective self‐management of T2DM.^18^ A recent meta‐analysis of studies conducted in developed nations found that patient‐centered self‐management interventions (PACE‐SMIs) incorporating both educational and behavioral components can significantly lower HbA1c and improve self‐care behaviors in adults with T2DM compared with the usual care.^19^ To determine its effectiveness in LMICs, this trial tested the efficacy of a PACE‐SMI to improve HbA1c, self‐efficacy, and self‐care behaviors in adults with T2DM in Pakistan.

## 2 METHODS

### 2.1 Study design and participants

A multicenter, parallel two‐arm randomized controlled trial was conducted at out‐patient departments (OPD) of four public‐sector tertiary hospitals in Faisalabad, the third largest city located in central Punjab Province of Pakistan. The participants were recruited by reviewing consecutive medical records and by inviting patients (who met eligibility from medical records) for a screening appointment. The inclusion criteria for participants were adults aged ≥18 years with a diagnosis of T2DM for ≥6 months. The exclusion criteria were T2DM patients with (1) HbA1c < 7, (2) uncontrolled psychological comorbidity (psychosis, schizophrenia, or severe learning difficulties), (3) severe comorbidity (cancer, stroke with disabilities, or need for regular dialysis which may limit their participation), (4) primary physician determined life expectancy of <6 months, (5) pregnancy, and (6) residence outside of the city which may impede home visit. Before implementing the trial, we conducted a pilot study to confirm the final design. This involved three phases: (1) need assessment in the context via direct observation and interviews with the key stakeholders, (2) development of PACE‐SMI based on social cognitive theory and a framework outlining justification, and the underlying assumptions, and (3) pilot testing of PACE‐SMI. The SPIRIT (Standard Protocol Items: Recommendations for Interventional Trials) compliant study protocol including PACE‐SMI description has been previously published elsewhere.^20^

The trial received ethical approval from Shifa Tameer‐e‐Millat University, Islamabad, Pakistan (IRB and EC no. 335–21) and Faisalabad Medical University, Faisalabad, Pakistan (ERC/FMU/2020–21/193). The written informed consent was obtained from all the participants through their signature and/or thumbprint. An independent data monitoring committee monitored collection, management, and analysis of the data. The trial has been conducted, analyzed, and reported according to the Consolidated Standards of Reporting Trials Guidelines for Social and Psychological Intervention (CONSORT‐SPI).^21^

### 2.2 Randomization and masking

A stratified permuted block (block size 4–6) randomization with the allocation concealment by opaque sequential numbered sealed envelopes was used to randomize recruited participants into the control group (CG) and the intervention group (IG). The randomization plan was provided by an independent data analyst to avoid any influence by the Principal Investigator (PI) or the research team members. The PI executed randomization plan by opening the sealed envelope in front of the participant and securing it with the serial number for audit and record purpose.

The nature of PACE‐SMI made it impractical to mask the study participants or the intervention administrators including the PI and research assistants (RA). However, to mitigate bias, the outcomes data were collected by the data collectors (DC) who were unaware of the participants' group assignments. The baseline data were collected prior to the randomization process to avoid any influence. To avoid data entry errors, an independent data entry operator was tasked to perform double data entry.

### 2.3 Procedures

The participants in the CG received usual care at the study hospitals' OPD which involved blood sugar measurement, a brief history, consultation with the physician, drug prescription and general information on lifestyle modification communicated either verbally or through written pamphlets. The participants in the IG received the same usual care, as well as nurse‐led PACE‐SMI. The PACE‐SMI was grounded in Social Cognitive Theory,^22^ and the contents were informed by American Association of Diabetes Educators self‐care behaviors.^23^ The detailed contents of PACE‐SMI are described elsewhere.^20^ The culturally tailored PACE‐SMI was delivered in eight weekly face‐to‐face individualized education, counseling and behavioral training sessions including a booster session, and a home visit. The educational sessions comprised of teaching the fundamental concepts related to the disease, its types, risk factors, treatment options, the role of self‐management through changing self‐care behaviors, and the provision of self‐care guidebook in the national language Urdu. The counseling sessions included verbal persuasion, discussion on initiating, and maintaining self‐care behavioral change, motivational video, and real‐time stories of patients with T2DM (who successfully managed their condition) to serve as the role models. The behavioral training sessions consisted of the six‐step behavior modification process aimed at enhancing self‐efficacy in four self‐care behaviors (1) diet control, (2) physical activity, (3) foot care, and (4) medication adherence. The booster session involved reflection on learning from the previous sessions, feedback on self‐care behaviors performance, a review of behavioral goals with personalized reinforcement fostering continued performance accomplishment, and a follow‐up plan. The home visit served two purposes: (1) observing and discussing personal, sociocultural, and environmental facilitators or barriers influencing initiation and continuation of self‐care behavioral change, (2) observing family and social support as a key strategy in initiating and maintaining behavior change. Each session lasted 60 to 90 min and was conducted by the PI with support from RAs in OPD room of the study hospitals. The PI, in‐charge of administering PACE‐SMI, was a registered nurse with a master's degree in nursing and a second master's in clinical psychology. The RAs were registered nurses who had completed 2‐day intensive training provided by the PI on the intervention delivery protocol and procedures assuring protocol compliance and consistency. A comprehensive manual of operations outlining the content, structure, delivery, and safety considerations was developed and followed to ensure quality and reproducibility. The checklists were maintained to assess the compliance with the intervention delivery protocol. The participants were followed up at 3 months following the intervention. The phone calls and frequent SMS reminders were used to ensure compliance with the behavior change during follow‐up period.

### 2.4 Outcomes

The HbA1c was assessed by collecting venous plasma samples for testing in a single central laboratory ensuring consistency in the methodology. The laboratory used was International Organization for Standardization (ISO) certified, and the College of American Pathologists (CAP) accredited. Before assessing this outcome, the DCs (who were registered nurses) had completed a robust 2‐day training provided by the PI on uniform and consistent procedure for venipuncture, sample collection, and delivery of the sample within 1 h of collection to the laboratory for testing.

The self‐efficacy was measured on a 20‐item Diabetes Management Self‐Efficacy Scale (DMSES)^24^ using its Urdu translated and validated version (U‐DMSES).^25^ Each item's response was rated on 11‐point scale, with a total possible score between 0 and 200.

The self‐care behaviors were measured on the Summary of Diabetes Self‐Care Activities (SDSCA) scale^26^ using its Urdu translated and validated version. The 15 items measuring diet, physical activity, foot care, and medication adherence were used. Each item's response was rated on a scale between 0 and 7, measuring the frequency of self‐care activities performed over 7 days' period with a total possible score between 0 and 105.

All outcomes were assessed at the baseline (T0), at the end of intervention (T1), and at 3 months' follow‐up (T2). The follow‐up assessments were obtained during an in‐person visit of the participant to OPD of the study hospital. A checklist was maintained for recording and reporting adverse events. The participants were encouraged to inform the PI of any adverse events. No adverse events or deaths were reported.

### 2.5 Statistical analysis

Based on the findings of a prior similar trial conducted in another country,^27^ we calculated the sample size using a priori power analysis with the G*Power 3.1 software. We set an alpha (*α*) level of 0.05, a power (1‐*β*) of 0.80 and an effect size of 0.23. This effect size was derived from the mean (Group 1 = 7; Group 2 = 7.3) and standard deviation (SD) (Group 1 = 1.2; Group 2 = 1.4) values reported in the trial.^27^ The G*Power calculation indicated that a sample size of 470 participants (235 in each group) would be required. To account for an estimated 30% dropout rate, we targeted a total of 612 participants (306 in each group) for this study.

The data were analyzed using Statistical Package for the Social Sciences (SPSS, version 25.0). The demographic and clinical characteristics of the participants were summarized using the mean (standard deviation [SD]) for continuous data and the count (percentage [%]) for categorical data. The differences in the baseline sociodemographic and clinical characteristics of the participants in the CG and the IG were analyzed using an independent *t*‐test (for continuous variables) and chi‐square test (for categorical variables). An independent *t*‐test was used to compare the difference in mean HbA1c, self‐efficacy, and self‐care behaviors between the CG and the IG at T0, T1, and T2 following confirmation of the normality. All tests were one‐tailed, at 95% confidence intervals (CI), considering a *p* value <0.05 as statistically significant. The rationale for using one‐tailed tests was to increase the sensitivity of analysis in detecting positive change aligned with the study hypotheses, as we anticipated that the PACE‐SMI would improve these outcomes based on the initial evidence provided by similar prior studies in the other contexts.

The Cohen's *d* was calculated to estimate the effect size to assess the magnitude of effect PACE‐SMI had on the HbA1c, self‐efficacy, and self‐care behaviors. In addition, linear regression analyses were performed to assess both the direct effect of PACE‐SMI on the HbA1c and the mediating effect of self‐efficacy and self‐care behaviors in this relationship. The first model evaluated the direct effect of the IG (CG vs. IG) on HbA1c at T2. The second model included self‐efficacy and self‐care behaviors as mediating variables to investigate their impact on changes in HbA1c levels.

In this study, both intention‐to‐treat (ITT) and per‐protocol (PP) analyses were considered. The ITT analysis includes all randomized participants, regardless of completion of follow‐up, providing a conservative estimate and reflecting real‐world scenarios. However, for the primary analysis, we employed a PP approach to evaluate the true efficacy of the PACE‐SMI among those who adhered to the study protocol. This approach helps to understand the potential benefits of full participation and reduces the risk of attrition bias. To address missing or incomplete data, we used interviewer‐administered questionnaires, double data entry by an independent data entry operator, and regular data quality checks to ensure accuracy and mitigate potential biases in the study results.

The trial was registered with ClinicalTrials.gov (NCT05491252) on August 8, 2022 after initial phase of study when the participants' recruitment was completed. The datasets generated during and/or analyzed in this trial are available from the corresponding author upon reasonable request.

## 3 RESULTS

**FIGURE 1:**
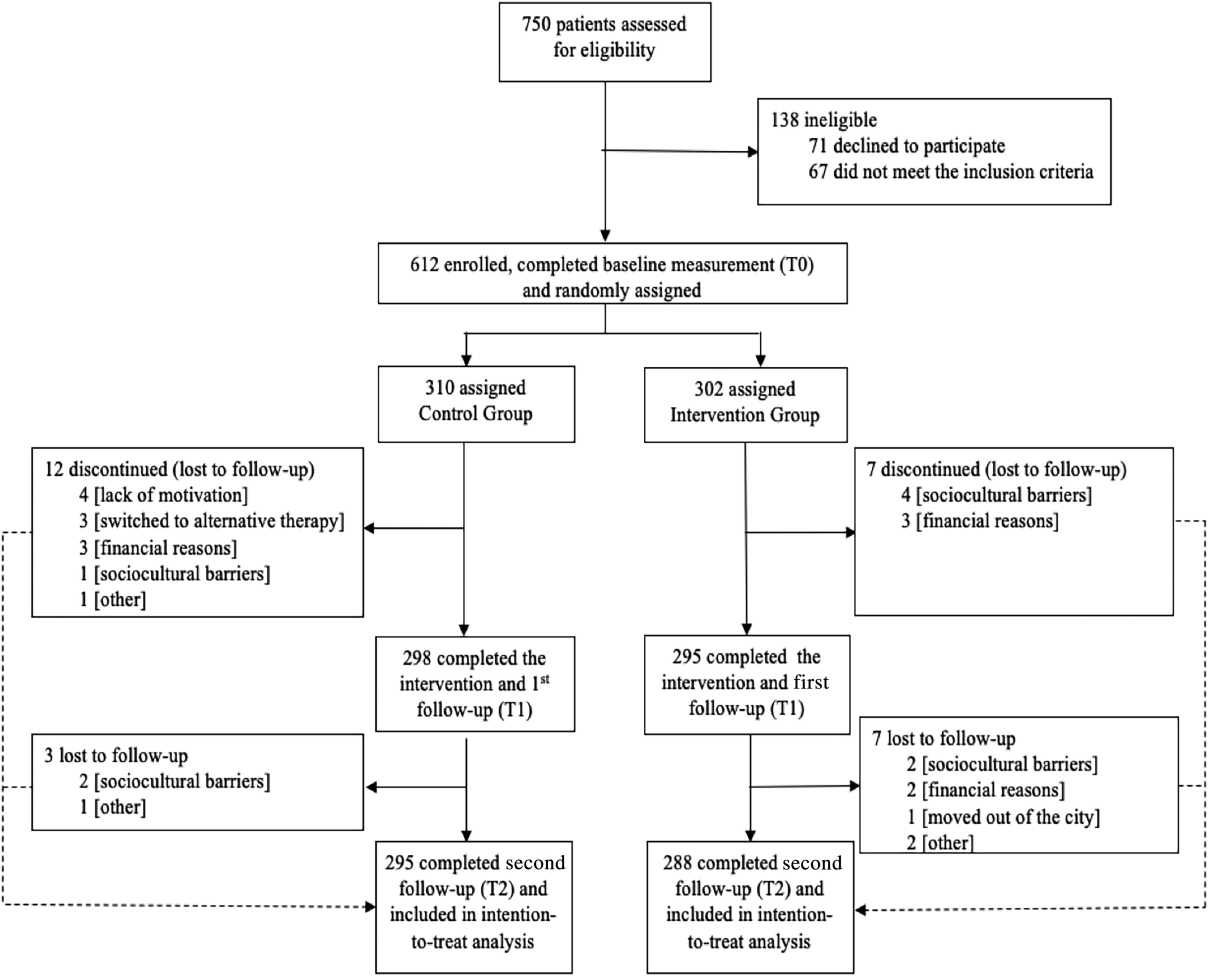
Trial profile

The trial profile in the Figure 1 shows that between April 21, 2022 and July 27, 2022, a total of 750 potential participants were assessed for eligibility. Overall, 138 were excluded (71 declined to participate; 67 did not meet the inclusion criteria) and 612 were randomly assigned to the CG (310), and IG (302). With a 4.74% (29/612) drop out; 583 participants were included in the PP analysis. The insignificant number of dropouts in both the groups were unlikely to affect the results of the trial; hence, further comparative analyses of variables between the participants who remained and those who dropped out were unwarranted.

The sociodemographic findings of the sample revealed that the age of the participants ranged from 31 to 78 years with mean age of 53.57 (SD, 9.45) years. There were almost equal proportion of men and women in the sample (51% and 49%, respectively). The majority had elementary to secondary school education (82%), mostly married (86%), living with their families (91%), and reporting very high family involvement in the care (90%). The clinical findings revealed that the mean duration of T2DM was 6.32 (SD, 4.69) years. The majority had a family history of DM (65%). Nearly half (47%) of the participants had one or more DM‐related complications. Most had major cardiovascular disease (CVD) risk factors including hypertension (78%), high cholesterol (48%), and smoking (43%). The sociodemographic and clinical characteristics of the participants in all four settings (A, B, C, and D) were similar. Likewise, the two groups were similar with regards to important sociodemographic and clinical characteristics at T0 (see Table 1).

**TABLE 1:** Baseline demographic and clinical characteristics of the participants (*n* = 612).

	Control group	Intervention group
	(*n* = 310)	(*n* = 302)
	*n* (%)	*n* (%)
Gender		
Male	169 (55)	144 (48)
Female	141 (45)	158 (52)
Education		
No formal education	25 (8)	19 (6)
Elementary to secondary	254 (82)	248 (82)
University education	31 (10)	35 (12)
Occupation		
Nonworking	152 (49)	143 (47)
Skilled workers	97 (31)	104 (34)
Businessmen/women	61 (20)	55 (19)
Marital status		
Single/other	18 (6)	17 (6)
Married	260 (84)	264 (87)
Widowed	32 (10)	21 (7)
Living arrangements		
Living alone	15 (5)	11 (4)
Living with family	281 (91)	275 (91)
Living with others	14 (4)	16 (5)
Family involvement in care		
No	33 (11)	28 (9)
Yes	277 (89)	274 (91)
Family history of DM		
No	109 (35)	107 (35)
Yes	201 (65)	195 (65)
Current treatment		
Insulin	89 (29)	84 (28)
Oral medications	175 (56)	173 (57)
Both	46 (15)	45 (15)
Complications		
None	165 (53)	157 (52)
One	117 (38)	124 (41)
More than one	28 (9)	21 (7)
Comorbidities		
None	61 (20)	57 (19)
One	182 (59)	183 (60)
More than one	67 (21)	62 (21)
Smoking history		
No	176 (57)	174 (58)
Yes	134 (43)	128 (42)
High blood pressure		
No	67 (22)	70 (23)
Yes	241 (78)	232 (77)
High cholesterol		
No	159 (51)	160 (53)
Yes	151 (49)	142 (47)
History of myocardial infarction		
No	269 (87)	264 (87)
Yes	41 (13)	38 (13)
Amputation		
No	304 (98)	297 (98)
Yes	6 (2)	5 (2)

	Mean (SD)	Mean (SD)
Age	53.87 (9.24)	53.25 (9.67)
Household income in PK rupees	30 277 (11 581)	29 063 (10 821)
	28 000 (Median)	28 000 (Median)
	25 000 (Mode)	20 000 (Mode)
Duration of disease	6.23 (4.72)	6.41 (4.68)

Abbreviations: DM, diabetes mellitus; PK, Pakistan; SD, standard deviation.

**FIGURE 2:**
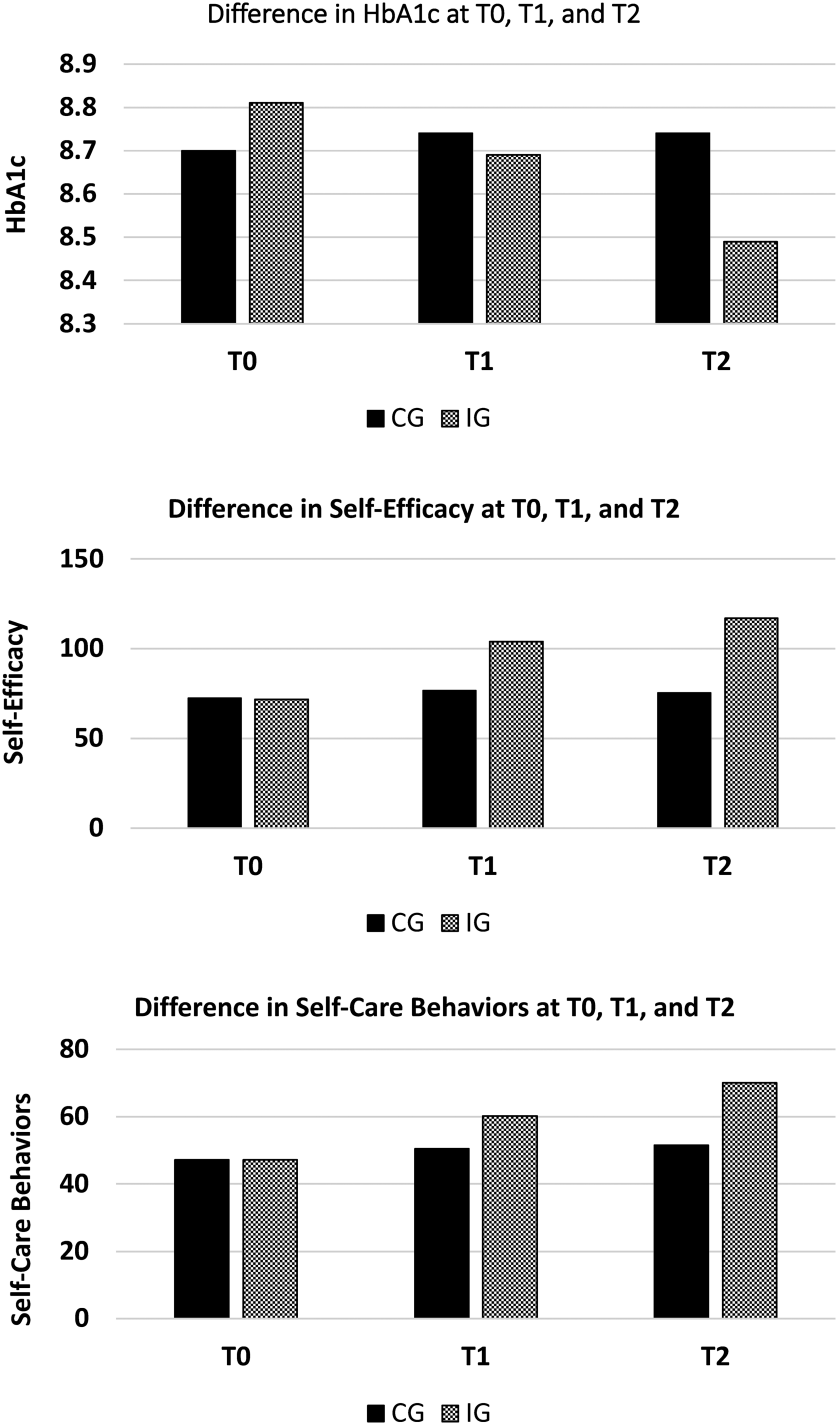
(A) Change in HbA1c at T0, T1, and T2; (B) change in self‐efficacy at T0, T1, and T2; and (C) change in self‐care behaviors at T0, T1, and T2. CG, control group; IG interventional group.

The outcomes data on the HbA1c, self‐efficacy, and self‐care behaviors at T0, T1, and T2 are presented in Table 2. This table shows that at T0, both the CG and IG were similar with regards to the HbA1c, self‐efficacy, and self‐care behaviors, indicated by no significant mean difference. At T1, no significant difference in the HbA1c was observed between the CG and the IG. However, at T2, difference in the HbA1c between the CG and the IG was small yet statistically significant (estimated mean difference, 0.25% [95% CI, −0.01 to 0.51]; *p* = 0.03). The results on the self‐efficacy indicate that at T1, mean scores of self‐efficacy significantly improved in the IG (estimated mean difference 27.28 [95% CI, 24.71–29.85]; *p* < 0.0001). Correspondingly, the difference in self‐efficacy between the CG, and the IG at T2 was also found statistically significant (estimated mean difference, 41.48 [95% CI, 37.83–45.13]; *p* value <0.0001). The results on the self‐care behaviors indicate that at T1, mean scores of self‐care behaviors significantly improved in the IG (estimated mean difference, 9.74 [95% CI, 8.09–11.39]; *p* < 0.0001). Likewise, the difference in self‐care behaviors between the CG and the IG at T2 was also found statistically significant (estimated mean difference, 18.56 [95% CI, 16.08–21.04]; *p* value <0.0001). The changes in the HbA1c, self‐efficacy, and self‐care behaviors at T0, T1, and T2 are shown in the Figure 2.

**TABLE 2:** HbA1c, self‐efficacy, and self‐care behaviors data at T0, T1, and T2.

	T0				T1				T2				
	CG (*n* = 310)	IG (*n* = 302)	Estimated mean difference (95% CI)	*p* value	CG (*n* = 298)	IG (*n* = 292)	Estimated mean difference (95% CI)	*p* value	CG (*n* = 295)	IG (*n* = 288)	Estimated mean difference (95% CI)	*p* value	Cohen's *d*
HbA1c	8.70 (1.48)	8.81 (1.65)	0.11 (−0.14 to 0.36)	0.38	8.74 (1.56)	8.69 (1.58)	0.05 (−0.20 to 0.30)	0.35	8.74 (1.62)	8.49 (1.58)	0.25 (−0.01 to 0.51)	0.03	0.16
Self‐efficacy	72.45 (15.11)	71.70 (13.64)	0.75 (−1.54 to 30.4)	0.26	76.51 (16.62)	103.79 (150.2)	27.28 (24.71 to 29.85)	<0.0001	75.43 (18.99)	116.89 (25.50)	41.48 (37.83 to 45.13)	<0.0001	1.84
Self‐care behaviors	47.09 (6.29)	47.14 (5.47)	0.05 (−0.89 to 0.99)	0.46	50.47 (7.52)	60.21 (12.33)	9.74 (8.09 to 11.39)	<0.0001	51.54 (12.04)	70.01 (17.97)	18.56 (16.08 to 21.04)	<0.0001	1.22

*Note*: Mean (SD) are reported for HbA1c, self‐efficacy, and self‐care behaviors, and independent *t*‐test has been applied.

Abbreviations: CG, control group; IG interventional group; SD, standard deviation; T0 baseline; T1, first follow‐up at the end of intervention; T2, second follow‐up at 3 months following T1.

To investigate the direct effect of PACE‐SMI on HbA1c, a linear regression analysis was performed, ensuring that all the assumptions were met. The results shown in Table 3 indicate that the model accounted for a small proportion of variance in the HbA1c levels (*R* = 0.079, *R* ^2^ = 0.006, adjusted *R* ^2^ = 0.005). However, this effect was not statistically significant (*p* = 0.056). This indicates a weak positive relationship between participation in PACE‐SMI and HbA1c reduction, suggesting that the intervention may have a modest effect on the glycemic control. Furthermore, to evaluate the mediating effect of the self‐efficacy and self‐care behaviors on HbA1c, a multiple linear regression analysis was performed. HbA1c at T2 was the dependent variable, while the IG (CG vs. IG), self‐efficacy, and self‐care behaviors at T2 served as independent/mediating variables. The results, detailed in Table 3, indicate that the model explained a significant proportion of variance in HbA1c levels (*R* = 0.482, *R* ^2^ = 0.232, adjusted *R* ^2^ = 0.228, *p* < 0.001). This suggests that participation in the PACE‐SMI was associated with a substantial reduction in HbA1c, mediated in part by the improvements in self‐efficacy and self‐care behaviors. These findings highlight the role of self‐efficacy and self‐care behaviors as potential mechanisms through which PACE‐SMI contribute to the improved glycemic control in adults with T2DM.

**TABLE 3:** Regression results for direct effect of PACE‐SMI and mediating effects of self‐efficacy and self‐care behaviors on HbA1c levels at T2.

Model	*R*	*R* ^*2*^	Adjusted *R* ^2^	Standard error of the estimate	Change statistics					
					*R* ^2^ change	*F* change	df1	df2	Sig. *F* change	Durbin–Watson
1	0.079^a^	0.006	0.005	1.6007	0.006	3.674	1	581	0.056	
2	0.482^b^	0.232	0.228	1.4092	0.226	85.301	2	579	0.000	1.780

Abbreviations: CG, control group; IG interventional group.

^a^ Predictors: (constant), group (IG vs. CG).

^b^ Predictors: (constant), group (IG vs. CG), self‐efficacy (SE_T2), self‐care behaviors (SCB_T2).

^c^ Dependent variable: HbA1c_T2.

## 4 DISCUSSION

To our knowledge, this is the first large‐scale multicenter trial in a LMIC, testing the efficacy of PACE‐SMI, a multicomponent, theory‐based, nurse‐led intervention to improve HbA1c, self‐efficacy, and self‐care behaviors in adults with T2DM. In this trial, the sample consisted of about equal numbers of men and women, with the mean age of 53.57 (SD, 9.45) years, mostly married, living with their families and reporting very high involvement of family in the care, mostly with limited education and financial resources, and high frequency of major CVD risk factors, such as hypertension, high cholesterol, and smoking. The findings indicated that compared with the usual care, PACE‐SMI resulted in modest yet statistically significant improvement in the HbA1c, as well as significant enhancement in the self‐efficacy and self‐care behaviors at 3 months. Our findings confirm that PACE‐SMIs previously tested in the developed countries are also effective in a LMIC, despite low level of reported education among the study participants.

The HbA1c is the key clinical indicator for optimum management of T2DM. The improvement in HbA1c in the IG who received PACE‐SMI in this trial was small yet statistically significant at 3 months. While the mean difference in HbA1c between both the groups appear modest, its clinical significance warrants careful consideration. The research has demonstrated that even small reductions in the HbA1c can lead to meaningful reductions in the risk of DM‐associated complications. For instance, the UK Prospective Diabetes Study (UKPDS) showed that a 1% decrease in HbA1c is associated with a 21% reduction in the risk of any DM‐associated complication.^28^ Although our study's mean difference of 0.25% in the HbA1c is less than the 1% threshold, it still represents a step toward reducing T2DM associated risks, especially when considered in the context of sustained long‐term management. Moreover, PACE‐SMI significantly improved self‐efficacy and self‐care behaviors, which are critical components of effective T2DM management. Our findings demonstrated that adults with T2DM who received PACE‐SMI had significantly improved self‐efficacy and self‐care behaviors compared with those in the CG. Importantly, the improvement in self‐efficacy and self‐care behaviors in our study lasted and was sustained at 3 months' follow‐up. These findings are contradictory to those reported by Manjula in her trial that initial improvement in self‐efficacy and self‐care behaviors faded overtime in the IG.^29^ The booster session and home visit in our trial involving personalized reinforcement fostering continued performance accomplishment and ongoing telephonic support via calls, as well as SMS reminders might have resulted in self‐efficacy and self‐care behavior change that was enduring. The literature supports that enhanced self‐efficacy has been linked to better adherence to the treatment regimens, improved glycemic control, and sustained lifestyle changes.^30^ The sustained improvements in self‐efficacy and self‐care behaviors suggest that the benefits of PACE‐SMI may extend beyond immediate reduction in the HbA1c, potentially leading to more substantial clinical outcomes over time. Although the reduction in HbA1c observed in our trial is small, it is statistically significant and clinically relevant, especially when considered alongside the notable improvements in self‐efficacy and self‐care behaviors. Additionally, the small but significant effect size of 0.16 indicates that PACE‐SMI had a measurable impact on the HbA1c. In the context of public health, even small effect sizes can be meaningful when applied to large populations, suggesting that PACE‐SMI could be beneficial on a broader scale, especially in the resource‐constrained settings. Especially, considering the challenges of T2DM management in LMICs such as Pakistan, where healthcare resources are often limited and disparities in care are prevalent, even modest improvements in the HbA1c are valuable. Our findings underscore the potential of the PACE‐SMI to make a positive impact on T2DM management in such settings, providing a feasible and effective strategy to improve patient outcomes.

Our study's findings on HbA1c reduction are contradictory to a prior trial conducted in Pakistan by Khan et al.,^17^ who found no significant reduction in HbA1c. Compared with their integrated diabetes care package involving educational component on lifestyle modification, our intensive 8‐week intervention with a patient‐centered approach grounded in Social Cognitive Theory, involving multiple components (education, counseling, and behavioral training), and covering sizable sample with negligible dropouts has a notable difference that resulted in small yet statistically significant reduction in HbA1c. These findings build on the existing evidence confirming that self‐management interventions involving behavioral components that go beyond the provision of education are more effective in T2DM management.^31^ The education alone equips patients with the essential knowledge about the disease and its management, but combining it with the counseling and behavioral training addresses emotional support and skill‐building, crucial for long‐term adherence, and behavior change.^11^, ^32^ Counseling helps individuals set realistic and personalized goals, addressing the psychosocial aspects of T2DM, which are often overlooked but are crucial for long‐term adherence to self‐care behaviors. Counseling can help patients overcome barriers to behavior change, cope with the emotional burden of the disease, and enhance motivation. Effective counseling has been associated with the improvements in self‐efficacy and a reduction in T2DM related distress.^11^ Behavioral training on the other hand, focuses on skill‐building and self‐regulation strategies, empowering participants to translate their knowledge and personalized goals into actionable behaviors. Research indicates that behavioral interventions can significantly enhance self‐care behaviors and glycemic control in patients with T2DM.^31^, ^33^ While each component—education, counseling, and behavioral training—plays a vital role in T2DM management, their combined implementation is crucial for achieving lasting behavior change and effective T2DM management. PACE‐SMI's success in significantly improving self‐efficacy and self‐care behaviors, sustained at the 3 months' follow‐up, underscores the importance of a multifaceted approach. These findings suggest that although education is fundamental, the addition of counseling and behavioral training can lead to more lasting changes in self‐efficacy and self‐care behaviors thereby leading to effective T2DM management.

The 3‐month follow‐up period in our study serves as an initial assessment of the effectiveness of PACE‐SMI, focusing primarily on short‐term outcomes. Although our findings indicate improvements in self‐efficacy and self‐care behaviors during this period, the sustainability of behavioral changes over the longer period remains a critical concern in T2DM management. Longer follow‐up durations are essential to evaluate the durability of intervention effects and to ascertain whether initial improvements in HbA1c, self‐efficacy, and self‐care behaviors persist over time. Future research should therefore prioritize extended follow‐up periods to better understand the trajectory of these outcomes. Such investigations would provide valuable insights into the lasting impact of PACE‐SMI and guide the development of strategies to maintain long‐term improvements in T2DM management.

In addition to examining the duration of follow‐up, the feasibility and scalability of implementing PACE‐SMI in Pakistan, amid significant healthcare access and resource disparities, demand pragmatic solutions to maximize its reach while minimizing the costs. The PACE‐SMI's emphasis on patient‐centered education, counseling, and behavioral training aligns with Pakistan's sociocultural fabric, where familial and community support plays a crucial role in health and disease management. Involving local healthcare providers, community leaders, and workers in PACE‐SMI's delivery can enhance its adoption across diverse socioeconomic groups, addressing healthcare disparities and building sustainable capacity within the local healthcare system. With modest training of healthcare workers and the integration of digital technologies, PACE‐SMI can be accessible and scalable, effectively bridging gaps in healthcare delivery while remaining cost effective.

Given the high prevalence of CVD risk factors among participants in our trial and the broader context of Pakistan, where hypertension, dyslipidemia, and tobacco use are significant public health concerns, there is a critical imperative to address these issues comprehensively. By integrating components focused on smoking cessation interventions, self‐management of blood pressure, and cholesterol control into PACE‐SMI, future studies can enhance its impact on reducing CVD risk among adults with T2DM in Pakistan. Emphasizing these components within PACE‐SMI could potentially mitigate the burden of CVD risk factors, offering a scalable and sustainable strategy for addressing complex health challenges in Pakistan.

### 4.1 Limitations

This trial has the following limitations: first, similar to the trials testing behavioral interventions, masking was not possible at all levels. However, to overcome the potential observer bias, alternative strategies such as the following were applied: data were collected on all participants before randomization, outcomes data were assessed by DCs unaware of the participants' group assignment, and an independent data entry operator did double data entry to minimize data entry errors. Second, outcome data on self‐efficacy and self‐care behaviors in this trial were self‐reported that might have carried response bias. The instruments we used to measure specific self‐care behaviors, such as diet control, physical activity, foot care, and medication adherence, may not offer precise estimation of behaviors performed. The differences in the participants' actual practices and the self‐report data they provided require further research.

### 4.2 Strengths

The strengths of the trial include a robust design, sufficient sample with high response rate, intensive multicomponent nurse‐led patient‐centered intervention, and high family involvement in the participants' care.

## 5 CONCLUSIONS

In this study, the PACE‐SMI demonstrated a modest yet significant improvement in the HbA1c, as well as notable enhancements in the self‐efficacy and self‐care behaviors in adults with T2DM. Given the significant health and economic impact of T2DM in Pakistan, these findings support the integration of PACE‐SMI into standard practice, providing critical guidance for the national healthcare policies.

## AUTHOR CONTRIBUTIONS

KA was the principal investigator; led formative phase, study design, and conduct of the study; did statistical analyses; and drafted the report. She assumes the responsibility for completeness and integrity of the data and fidelity of the report to the study protocol and statistical analysis plan. ESF contributed to the scientific rigor of the study by contributing to all phases of this trial. She provided technical oversight of all project activities including coordination of data management and data analysis. KAD contributed to the study design, intervention development, and supervision of all project activities. RG contributed in study design, intervention development, and supervision of all project and budget activities. NK provided the randomization and statistical analyses plan and contributed to the interpretation of study findings. All authors had the opportunity to review and revise the report and approved the final submitted version of the manuscript.

## FUNDING INFORMATION

This trail was partially funded by Shifa Tameer‐e‐Millat University, Islamabad, Pakistan (grant no. 15270). The funder had no role in the design, data collection, analysis, interpretation, or writing the report.

## CONFLICT OF INTEREST STATEMENT

The authors declare no conflicts of interest.

## CLINICAL TRIAL REGISTRATION

The trial was registered with ClinicalTrials.gov (NCT05491252) on August 8, 2022.

## Data Availability

The datasets generated during and/or analyzed in this trial are available from the corresponding author upon reasonable request.
